# Annotations

**Published:** 1891-06-27

**Authors:** 


### Annotations.

The Sunday Collections.

The collections taken on Hospital Sunday have a trick of halting on the way from the church to the hospital treasury. They come with more or less of a rush during the first week; and after that they drop in one by one like the stragglers of a defeated army. But they do come sooner or later; and it has generally happened during the last few years that the final total of each succeeding year has been somewhat in advance of its predecessors. Let us hope that this year will prove no exception to the rule. The Reverend Dr. Forrest, now Dean of Worcester, but Vicar of St. Jude's, Kensington, on the date of the Sunday collection, has sent in the splendid sum of £1,300, the largest collection ever taken in any one church since the inception of the Fund. Canon Fleming is hardly second to Dr. Forrest in the amount contributed by St. Michael's, Chester Square, which is hard upon the thirteen hundred pounds of St. Jude's. Many other churches have done well this year; but some a good deal less than well. It is supposed that the 'bus strike operated unfavourably on the collections at St. Paul's and Westminster Abbey and at some other churches. Any excuse is better than none; but the Sunday Fund would much rather have had strong collections than weak apologies. An analysis of the amounts taken and of the subscribing congregations brings out the fact that the Church of England contributes an amount very disproportionate to that raised by the various nonconforming Churches. Now we do not expect that churches which have no endowments, and whose adherents in some instances at any rate, belong largely to the poorer classes, can afford to give nearly so much to a public object like the hospital collection as the wealthier members of a large and splendidly endowed organisation like the Church of England. But we do expect, and we have a right to expect, that many nonconforming congregations shall give a good deal more than they do, and that some who have hitherto given nothing at all shall now begin to give. Nonconformity this year seems to cut almost a poorer figure than ever. We are aware that some Nonconformists have made a grievance out of certain arrangements that prevail in the nursing departments of one or two hospitals. But to visit all the hospitals with punishment because one or two fail to reach a high standard of political wisdom is anything but wise, and it is certainly not Christian. Nonconformists profess to hold the pure faith of Christ, unmixed with worldly adulteration. It is a high claim they make upon the public intelligence: a claim of wisdom, largeness of mind, and forgiving generosity, and not merely of purity of doctrine. Few people will allow their claim in theory if they do little that corresponds with such a claim in actual practice. We have no wish to say one word against Nonconforming churches; but the "Dissidence of Dissent" ought not to choose Hospital Sunday and hospital work generally for a special display of its activity. "The unity of the spirit" might that day very well be the most marked characteristic of Christian activity and worship.

The General Practitioners' Union.

The general practitioners of medicine are finding it so difficult to live that they have formed a union amongst themselves with the object of trying to accomplish by combination that which they have failed to effect separately. It seems that there is in America a condition of things among family physicians similar to that which prevails in this country. There, as here, the voluntary hospitals compete for patients with those doctors who practise among the poor, to the entire discomfiture of the unfortunate private practitioner. "This wholesale free treatment of patients has," says the *New York Medical Journal*, "a demoralising tendency, making paupers of a large percentage of our population, and robbing the medical profession of a source of income which by right belongs to it. Further, it makes the road for the hard-working, industrious, but impecunious physician the more difficult to travel, and in time it will annihilate his existence. The mischief wrought by making any class of persons dependent, and thus destroying the spirit of self-reliance and independence, is not to be underestimated. If a man, able to work and earn a livelihood, is made to feel that he may have free medicine and medical treatment, it will not be long before he grows to think that he ought to be supplied with raiment and food free of charge. He will soon look upon himself as a favoured individual to whom the world owes a living, and will forget that the first condition of existence is labour." It is but too evident that the poorer part of the population, in English-speaking countries at any rate, have come to consider that they are entitled to free medical treatment. It is unhappily, also, all too manifest that physicians and surgeons are willing, for the sake of holding hospital appointments, to treat without too much scrutiny all who choose to make application. The practitioners who do not hold hospital appointments, and who have, therefore, nothing to gain but a good deal to lose by the undue extension of hospitals, naturally resent what they consider an unbrotherly use of accidental advantages. The General Practitioners' Union which has just been formed has a difficult problem to solve in the hospital question. It has our cordial sympathy in its arduous and important work.

Clerical Specialism.

Certain persons have made it an objection against the Bishop-designate of Lichfield that he is not a man eminent in any particular department of learning, and that he did not in early life distinguish himself at his University above most of his contemporaries. To this a country clergyman, who is opposed in politics to the new Bishop, makes reply in a morning paper thus: "As a neighbour of Canon Legge, with ample opportunities for estimating his work and character, I know no clergyman who in the days in which we live, possesses more fully the needful qualities for a spiritual overseer than the new Bishop of Lichfield. He may take for his motto the famous words of the Roman dramatist, 'Christianus sum, nihil in rerum naturâ a me alienum puto.' It is not required of a Bishop in these days that he should be a consummate scholar or a brilliant rhetorician, but it is required that he be large-minded and large-hearted, devout, humble, rejoicing to welcome truth and goodness wherever he finds them; and if he has a large fund of practical wisdom, untiring laboriousness, and a great capacity for getting through work, so much the better. I confidently appeal to all who know him best, to men of all parties and conditions, whether the new Bishop of Lichfield is not such a man." There is a limited class of self-asserting and foolish persons, who think they themselves have the first claim to promotion of every sort. This class consists largely of specialists. At college, a man has a great power of cram, and probably, also, in many cases is really a clever fellow. He distinguishes himself as a matter of course, and thereupon he assumes that he has established a claim to take precedence of all other kinds of people for the rest of his life. He is to be made head master of his own school, although he may have no more teaching capacity than a ploughman; and he is to be appointed Bishop of his diocese, in spite of the fact that he has no religious earnestness and no governing capacity. The same holds good of other professions. A particular medical student will cram up his anatomy and chemistry in quite an amazing way, and will carry all before him in Materia Medica and book midwifery. He thence concludes that he is bound to be either a distinguished physician or an eminent surgeon. As a matter of fact, he may have no diagnostic or operating capacity as a surgeon, and none of the reasoning power, or of the instincts and intuitions of the physician, and may, therefore, be neither physician nor surgeon in the true sense. This practice of taking academic distinction and specialism at its own valuation is becoming a little too absurd. In a practical world like our own we want to give a man those particular duties which he has proved himself fit for in actual practice. The Dry-as-dust must be relegated to his study and to his beloved particles; the practical ruler of men and the earnest religious teacher must be appointed to the office of Bishop. So also it should be in medicine. The man who can operate with skill, and not the mere crammer of books, must be made surgeon; and he who can apply himself with experienced resource and success to the practical relief of suffering and the cure of disease must be made physician, and not the mere man of the library, or the microscopic market gardener, whose proper business in life is the cultivation of minute fungi. Carlyle has told us repeatedly, and with all possible emphasis, that the great business of every department of life is "to give the tools to him who can handle them." To our thinking, the qualifications of the new Bishop of Lichfield, as set forth by his clerical neighbour, are the very gifts which a Bishop should possess. What we desire is that the tall soldier shall have the big uniform, and the short man the small one, whatever may have been the respective sizes of the two in their schoolboy days.

